# Research on corn production efficiency and influencing factors of typical farms: Based on data from 12 corn-producing countries from 2012 to 2019

**DOI:** 10.1371/journal.pone.0254423

**Published:** 2021-07-09

**Authors:** Jiamei Wang, Xiangdong Hu

**Affiliations:** Institute of Agricultural Economics and Development, Chinese Academy of Agricultural Sciences, Beijing, China; Universidad Nacional Autonoma de Nicaragua Leon, NICARAGUA

## Abstract

Globally, corn is characterised by high production and high export concentrations, yet the world is experiencing an unprecedented, huge change in this regard. Ensuring the global supply of corn, and thereby the energy and food security of nations has become particularly important. To understand the importance of corn production as an influencing mechanism of global food supplies, the present study researched the corn production of typical farms in major corn-producing and importing countries around the world. I selected the corn input and output data of 18 typical farms in 12 countries from 2012 to 2019, used the data envelopment analysis (DEA) model to calculate the technical efficiency of corn production, and built a tobit model to explore the impact of farming methods, input elements, supporting services, and other factors on efficiency. The study established that the average comprehensive technical efficiency of corn production on a typical farm was 0.863, and the average loss was 13.7%. In addition, it concluded that intensive tillage and conservation tillage have high technical efficiency. It also demonstrated that the proportion of mechanical labour and technical efficiency is in a ‘U’-shaped relationship, among others.

## Introduction

Corn is one of the most widely-planted crops in the world. It is grown in more than 170 regions globally. Corn production is highly concentrated in certain regions like North America, Asia, and South America. According to the United States Department of Agriculture, in 2020, corn production in the United States (US), China, Brazil, and Argentina accounted for 64.63% of global production [1]. In addition to holding inventory, a portion of the corn produced is consumed domestically, while the rest is exported. Corn exports and production are also highly concentrated. The main corn exporting countries are the US, Brazil, Argentina, and Ukraine. During 2020–2021, the cumulative corn exports of these four countries accounted for 88.12% of global exports [1]. This indicates that, although China is a major corn producer, it is not a major corn exporter. Global corn production is showing a slight downward trend, and the growth rate of consumption is higher than that of production. Global corn consumption is also highly concentrated. The US and China are the two largest corn consumers. In 2020, China’s corn consumption reached 279 million tons, an increase of 2 million tons from the previous year [1]. In recent years, the destocking speed of corn in China has accelerated, and nearly 260 million tons of stock have been consumed in 4 years [2]. With the continuous consumption of temporarily-stored corn, the overall corn supply has tightened, and the gap between supply and demand has gradually increased. According to UN Comtrade, China’s corn imports reached 11.3 million tons in 2020, a yearly increase of 135.73% [3]. China is a large corn consumer, most of which is from domestic production and a small part of it is from imports, but the import volume is showing an upward trend. Ukraine and the US are the main sources of China’s corn imports. In 2020, China’s corn imports from these two countries accounted for 94.20% of the total imports [3].

Grain production has achieved bumper harvests for 17 consecutive years, since China attaches great importance to food security, corn production has increased steadily in the past five years. However, there are still major problems in China’s corn production. From the perspective of supply and demand, the rapid development of animal husbandry has caused China’s corn consumption to exceed corn production since 2016, resulting in a large amount of imported corn. According to UN Comtrade, in terms of imports, corn reached 3.52 million tons in 2018, a yearly increase of 24.38%. In 2019, corn imports reached 4.79 million tons, a yearly increase of 36.08%. Simultaneously, since 2008, corn exports have reduced to 270,000 tons, a yearly decrease of 94.5%. Corn exports have been below 300,000 tons in the past 12 years [3]. Regarding production costs, China’s demographic dividend period has passed, and labour costs have increased significantly; land costs, seeds, fertilisers, and other agricultural materials costs have also increased, increasing the total cost of corn. From a pecuniary point of view, due to China’s policy of supporting the food market for many years, coupled with the increase in corn production costs, the phenomenon of domestic and foreign corn prices is inverted. Effectively ensuring corn production, increasing corn productivity, and ensuring food security have become particularly important. This study examines the production technology efficiency of 12 major corn-producing countries around the world from a macro perspective, analyses the main influencing factors, explores its influence mechanism, and proposes policy recommendations for improving China’s corn production capacity.

## Literature review

Agricultural growth mainly depends on the improvement of production efficiency, which is a key indicator of agricultural progress [4]. Improvements in technical efficiency are necessary to increase food production and release production potential [5]. To effectively improve technical efficiency, it is required to accurately estimate technical efficiency, analyse important factors affecting corn production efficiency, and propose targeted policy recommendations [6]. This article reviews the research on the technical efficiency of corn and other important agricultural products. One is to compare and sort out the methods of measuring efficiency in existing research, the other is to provide reference for the methods used in their own research programs, and the third is to compare and verify the existing research results with their own conclusions. Many scholars use certain methods to measure the technical efficiency of corn production, which can be roughly divided into two categories: parametric and non-parametric methods. One is to use the non-parametric data envelopment analysis (DEA) method to measure technical efficiency. Zhang, Meng and Gao [7] calculated the average technical efficiency of corn production in the Xiliao River Basin to be 0.88, based on the DEA of investment-oriented BCC model. They established that, compared with the traditional technology, the use of the non-film shallow drip irrigation technology improves the technical efficiency. Gao, Liu and Dai [8] conducted efficiency calculations on nine major corn-producing areas in Xinjiang in 2006. Two output indicators and two input indicators are used. The output indicators are mainly corn yield and output value per unit area. The input indicators use the working price and material cost per hectare in turn. Pei and Zhou [9] used the DEA method to evaluate corn production efficiency in Heilongjiang Province in 2014. The study established that the overall efficiency of corn production in Heilongjiang was 78.80%. Koc, Gul and Parlakay [10] measured through the DEA model that the technical efficiency of corn farms in the Eastern Mediterranean region of Turkey is 81%. Mulwa, Emrouznejad and Muhammad [11] used the DEA model to measure the technical efficiency of corn production in western Kenya and determined that the production efficiency was only 67.70%, which is technically inefficient. However, agricultural training can reduce this inefficiency. The extension agents organize farmer training sessions to inform of farmer field schools to inform farmers about modern farming methods. DEA is used to model efficiencies as an explicit function of seed, fertiliser, family labour, hired labour and hired capital costs. This study constructs a meta-frontier for the two regions.

Another way is to use a parametric method to measure technical efficiency using the stochastic frontier model. Based on the stochastic frontier analysis, at the end of the 20th century and the beginning of the 21st century, the average technical efficiency of corn production in China was above 0.8 [12, 13]. Moreover, there are obvious gaps in technical efficiency between regions, and the degree of technology utilisation is different [14]. The application of scientific research and development in corn production has an advancing effect on corn production, and technological progress is conducive to improving the efficiency of corn production technology [15]. By 2015, technical efficiency had slightly improved, stabilising above 0.9 [16]. Abdallah and Awal [17] reported that the technical efficiency of Ghana’s corn production from 2001 to 2004 was 53%. A Cobb-Douglas production function was used as the functional form of the stochastic frontier production function to define the relationship between outputs and inputs. Chiona, Kalinda and Tembo [18] conducted a stochastic frontier analysis of the technical efficiency of smallholder corn growers in the central province of Zambia and measured an average technical efficiency of 50%. This study specifies the stochastic frontier production function using the flexible translog specification. A likelihood test was conducted that the translog stochastic frontier production function can be reduced to a Cobb Douglas. Using the stochastic frontier method, Siaw et al. [19] measured the average technical efficiency of corn production in Ghana to be 74% and established that agricultural credit can increase technical efficiency by 8%. Some scholars use DEA and stochastic frontier method to measure the technical efficiency of corn production at the same time, and find that the efficiency calculated by DEA method is higher than that by stochastic frontier method. Hassan et al. [20] measured the technical efficiency of corn production in Nigeria from 1971 to 2010. The efficiencies measured by the stochastic frontier method and DEA were 64.1% and 87.7%, respectively. The average level of corn production efficiency in Indonesia calculated by Asmara using the stochastic frontier method was 0.78, and the technical efficiency calculated using the DEA method was 0.91 [21].

Technical efficiency measurement of other important agricultural products. The above summarizes the measurement of the transnational technical efficiency of corn. Some scholars have also measured the technical efficiency of other agricultural products. Here I mainly review the measurement of the efficiency of soybean, wheat, cotton, and coffee bulk agricultural products. For soybean varieties, some scholars use the super-logarithmic stochastic frontier model to measure the efficiency. Otitoju and Arene [22] measured the technical efficiency of soybean production in Benue State, Nigeria as 0.73, and Asodina et al. [23] measured 0.58 in Upper West Region, Ghana. For wheat varieties, Tuna and Oren [24] used DEA to measure the technical efficiency of wheat production in south-eastern Anatolia, Turkey to be 0.78. Some scholars use the stochastic frontier production function to measure the efficiency of wheat production technology. Jaime and Salazar [25] measured the efficiency of Chile as 0.6, and Kamruzzaman and Islam [26] measured the efficiency of Dinajpur District, Bangladesh as 0.7. Ojo [27] measured the technical efficiency of food crops in Swaziland to be 0.77. Some scholars used DEA to measure the technical efficiency of other agricultural products. Poudel, Yamamoto and Johnson [28] used DEA to measure the technical efficiency of coffee production in Nepal as 0.83. Gul [29] measured the cotton technical efficiency in Turkey to be 0.89. The above-mentioned scholars’ technical efficiency measurement and use methods of corn and other agricultural products in different regions are shown in Table 1. More about the measurement method of efficiency, from the beginning of Farrell to the improvement and innovation of the original method by later scholars, is presented by Zuniga who carried out a detailed and systematic combing [30].

**Table 1:** Technical efficiency of agricultural commodities in various regions and measuring approaches.

Author	Region	Commodity	Approach	Efficiency
Zhang, Meng and Gao (2020)	The Xiliao River Basin	corn	DEA	0.88
Gao, Liu and Dai (2008)	Xinjiang Province	corn	DEA	0.46
Pei and Zhou (2017)	Heilongjiang Province	corn	DEA	0.79
Koc, Gul and Parlakay (2011)	Turkey	corn	DEA	0.81
Mulwa, Emrouznejad and Muhammad (2009)	Kenya	corn	DEA	0.68
Kang and Liu (2005)	China	corn	SFA	0.81
Zhao and Jia (2011)	China	corn	SFA	0.82
Liu et al. (2018)	China	corn	SFA	0.90
Abdallah and Awal (2017)	Ghana	corn	SFA	0.53
Chiona, Kalinda and Tembo (2014)	Zambia	corn	SFA	0.50
Siaw et al. (2020)	Ghana	corn	SFA	0.74
Hassan et al. (2014)	Nigeria	corn	DEA	0.88
Hassan et al. (2014)	Nigeria	corn	SFA	0.64
Asmara et al. (2016)	Indonesia	corn	DEA	0.91
Asmara et al. (2016)	Indonesia	corn	SFA	0.78
Otitoju and Arene (2010)	Benue State, Nigeria	soybean	translog stochastic frontier model	0.73
Asodina et al. (2021)	Upper West Region, Ghana	soybean		0.58
Tuna and Oren (2006)	South-eastern Anatolia, Turkey	wheat	DEA	0.78
Jaime and Salazar (2011)	Chile	wheat	stochastic frontier production function	0.60
Kamruzzaman and Islam (2008)	Dinajpur District, Bangladesh	wheat		0.70
Ojo (2015)	Big bend, Swaziland	food crop		0.77
Poudel, Yamamoto and Johnson (2011)	Nepal	coffee	DEA	0.83
Gul (2009)	Turkey	cotton	DEA	0.89

Numerous scholars have researched the factors affecting corn production efficiency from different perspectives. Miho [31] studied corn planting efficiency in two areas of Tanzania and established that there is a positive relationship between the number of people who can work in the family and technical inefficiency. Production input inhibits the improvement of technical efficiency, and good living conditions can promote efficiency. Olarinde [32] established that the main determinants of the technical efficiency of maize planting in Nigeria include extension services, farming experience, and farm distance. Boundeth, Nanseki and Takeuchi [33] established that the technical inefficiency of corn growers in the northern provinces of Laos decreases with an increase in farm size. When studying the factors affecting corn production technology in China, Liu et al. [16] determined that the power of agricultural machinery has a positive effect on the technical efficiency of corn production and that the basic conditions of agriculture will affect the loss of technical efficiency. They proposed an increase in machinery investment to promote mechanised production. Wang and Wu [34] established that the use of biochemical inputs such as chemical fertilisers and the use of mechanical agricultural materials improved the technical efficiency of corn production and proposed increasing chemical fertiliser subsidies and promoting large-scale production. Jia and Xia [35] established that the scale efficiency and the corn planting area indicated an ‘inverted U-shaped relationship’. It can be seen that promoting the scale operation of grain is not the bigger the better. The government should give the guidance and standardization to farmers and make them seek the appropriate degree of scale operation.

Based on the above literature, it can be determined that many scholars use non-parametric analysis methods to measure efficiency in addition to parametric analysis methods. The parametric analysis method has higher requirements for the correct construction of the model and the selection of variables. Scholars have mostly used the envelope analysis method and linear programming to measure efficiency. Concurrently, it can also be established that most scholars focused on the perspective of corn production in a certain country, region, province, or state. They rarely explored the technical efficiency and factors influencing corn production across multiple countries. Therefore, in this study, the corn input and output data of 18 typical farms in 12 countries worldwide from 2012 to 2019 were selected. I used the DEA model to measure technical efficiency and the tobit model, considering the measured technical efficiency as the dependent variable, to explore the impact of the farming system, business scale, and quantity and structure of factor inputs on the efficiency of corn production technology.

## Materials and methods

### Model settings

#### DEA model

From an economic perspective, technical efficiency refers to the degree to which the production process of a production unit reaches the technical level of the industry, reflecting the technical level. In this study, DEA was used to measure technical efficiency. The DEA analysis method was proposed by Charnes, Cooper, and Rhodes in 1978; therefore, the first DEA model was named the CCR model. The CCR model assumes that the return to scale remains unchanged, and the measured technical efficiency includes scale efficiency, which is called comprehensive technical efficiency. However, in the actual production situation, the production scale of the production unit may not be optimal; therefore, this study chooses the BCC model based on variable returns to scale proposed by Banker et al. in 1984. Further, comprehensive technical efficiency is decomposed into pure technical and scale efficiency. For more introductions and extended applications of DEA, Dios-Palomares [36] and Zuniga [37] have thoroughly detailed their combing and practice. Unlike industrial production, which can control input and output, agricultural production can only control and adjust the input. Therefore, the input-oriented BCC model is selected, and the planning formula is as shown in formula (1):

$$\begin{array}{l} min\theta \\ s.t.\sum_{j=1}^{n}\lambda_{j}x_{ij}\leq \theta x_{ik}\\ \sum_{j=1}^{n}\lambda_{j}y_{rj}\geq y_{rk}\\ \sum_{j=1}^{n}\lambda_{j}=1\\ \lambda \geq 0\\ i=1,2,\ldots ,m;r=1,2,\ldots ,q;j=1,2,\ldots ,n \end{array}$$ (1)

**Fig 1:**
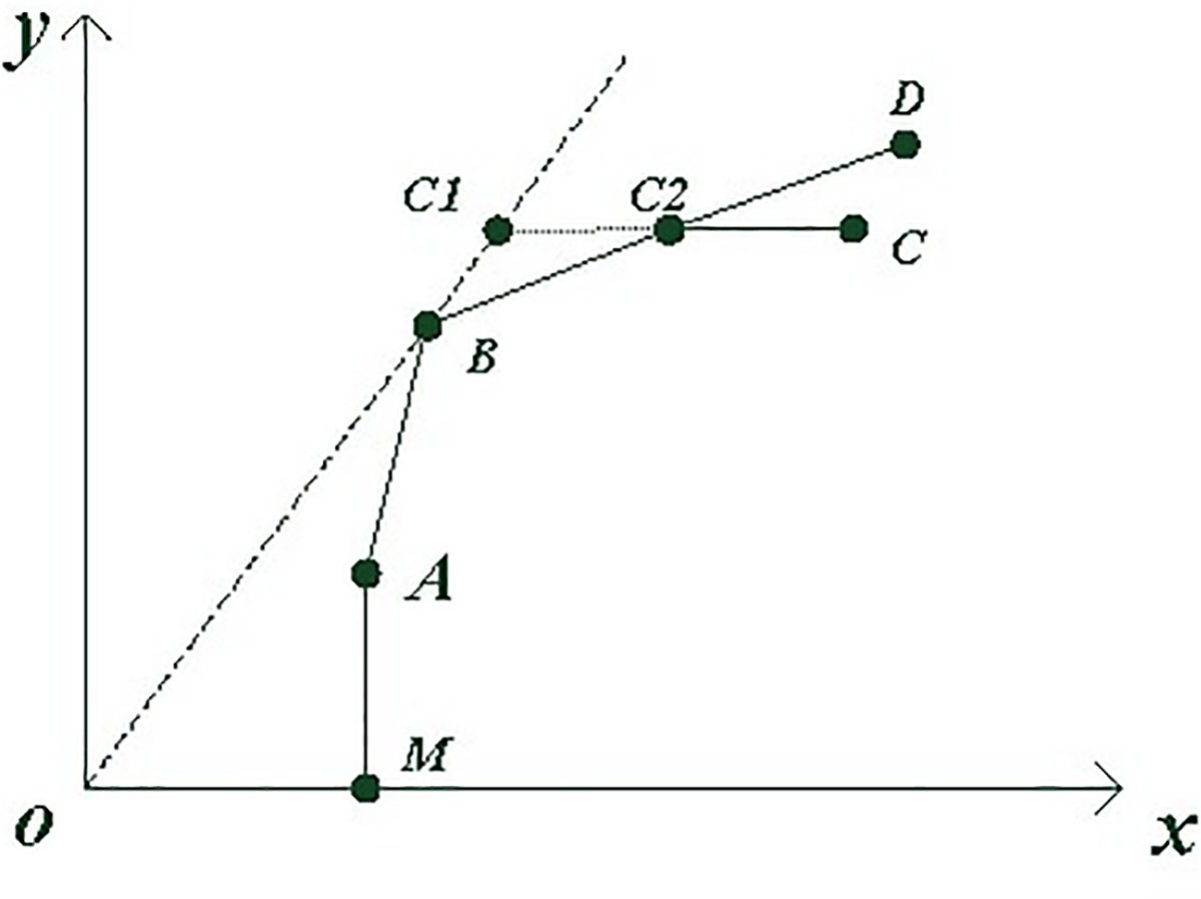
Diagram of the basic principle of the investment-oriented BCC.

The production unit that needs to be measured in DEA is called a decision-making unit (DMU). There are *n* units that measure efficiency, denoted as DMU_*j*,_ each of which has *m* types of inputs denoted as *x*_*i*_, and *q* types of outputs denoted as *y*_*r*_. The current DMU to be measured is denoted as DMU_*K*_, and the linear combination coefficient of the DMU is represented by λ. With a certain input, technical efficiency is the ratio of the actual output of a production unit to the production frontier. Under the assumption that the return to scale is constant, the production frontier can be represented by the OB ray in Fig 1, and B is the only effective production unit. In the case of variable returns to scale, the production frontier is a curve formed by MABD that is convex to the left.

#### Tobit model

To further study the influencing factors of technical efficiency, the technical efficiency measured by DEA was used as the dependent variable, and the influencing factors were regressed. As the efficiency value of a typical farm calculated by DEA is truncated data between 0 and 1, estimating it using the ordinary least squares method will cause bias and inconsistency. The tobit model can effectively reduce the deviation [38, 39] and is suitable for analysing the factors influencing technical efficiency [40]. Therefore, this study uses the tobit model, which is expressed as formula (2), and the maximum likelihood estimation method is used for the regression analysis. The data used in this study are from 2012 to 2019, which are short panel data. Due to the lack of sufficient statistics for individual heterogeneity, the fixed-effects tobit model cannot perform a maximum likelihood estimation, and the regression results are usually biased [41]. The use of the random effects estimation is effective [42]; therefore, this study adopts the tobit model of random effects.


$$\begin{array}{l} y_{it}^{*}=\beta_{0}+\beta x_{it}+\varepsilon_{it}\\ y_{it}=\left\{ \begin{array}{c} 0,y_{it}^{*}\leq 0\\ y_{it}^{*},0<y_{it}^{*}<1\\ 1,y_{it}^{*}\geq 1 \end{array}\right. \end{array}$$ (2)

In formula (2), $y_{it}^{*}$ is the typical farm efficiency vector, *β*_0_ is the intercept vector, ***β*** is the parameter vector to be estimated, ***x***_***it***_ is the explanatory variable vector, *ε*_*it*_ is the random disturbance item, and *y*_*it*_ is the restricted explained variable.

### Data source

The data used in this study are sourced from agri-benchmark, a global, non-profit network of agricultural economists, advisors, producers and specialists. It is managed by the Thünen Institute and global networks under the German Federal Ministry of Food and Agriculture. According to agri-benchmark, the standard definition of a typical farm is that a region or country has medium-scale and large-scale farms to reflect the average management level of most farms, that is, the average profit level. The areas where the selected typical farms are located are the intensive and main production areas of a particular crop. The cost-benefit data of a typical farm are collected in a comprehensive group with the participation of farmers and consultants, and standard questionnaires are issued to ensure that each figure reflects a typical situation.

This study selects corn input and output data of typical farms in 12 countries, that is, Argentina, Brazil, and Uruguay (South America), Russia, France, Ukraine, Bulgaria, Poland, Czech Republic, and Hungary (Europe), as well as the US (North America) and South Africa (Africa) including 18 typical farms such as AR330ZN, AR700SBA, AR900WBA, BR65PR, BR1300MT, US700IA, US1300ND, etc. from 2012 to 2019. The first two digits of the farm code represent the country, the number represents the size of the farm, and the last few alphabets represent the area where the farm is located. Consider US700IA as an example, which means a 700-hectare farm in Iowa, USA.

### Variable selection and descriptive analysis

#### DEA model

Based on the scholarly research of Yang and Lu [43], Zhao, Wang and Zhang [44], and Xiao and Zhao [45], this study selected corn yield per unit area as the output indicator, and land, labour, machinery and fuel, construction costs, and other miscellaneous expenses as input indicators. The land input is the corn planting and operating area of the farm, and the labour input is the amount of labour per unit area of corn production. Machinery and fuel inputs are machinery and fuel costs, and construction costs include depreciation, repair, and financial costs. Other miscellaneous expenses include inventory insurance premiums and taxes, consulting fees, and accounting costs. A descriptive analysis of the corn input and output indicators for typical farms is presented in Table 2.

**Table 2:** Descriptive analysis of corn production input and output indicators in typical farms.

Variable	Unit	Mean	Standard deviation	Minimum	Maximum
Output					
Crop yield	Tons/ha	8.13	2.96	1.49	14.00
Input					
Acreage	ha	529.51	690.08	10.00	4100.00
Seed	Tons/ha	17.58	9.09	0.91	30.60
Fertilizer	Tons/ha	234.63	201.06	68.94	1055.21
Pesticides	USD/ha	72.39	30.42	19.65	150.76
Labour	Person/ha	13.40	11.84	<0.01	56.84
Mechanical	USD/ha	174.87	151.33	<0.01	671.64
Fuel	USD/ha	244.55	172.85	53.43	846.23
Buildings	USD/ha	34.60	52.44	<0.01	281.02
Miscellaneous	USD/ha	63.27	71.50	1.36	354.93

#### Tobit model

Liu et al. [46] used the comprehensive technical efficiency measured by DEA as the dependent variable and land, machinery, and seed prices as independent variables to study the factors affecting corn production efficiency. They established that land cost, machinery cost, and seed price affect comprehensive technical efficiency. All of these factors have a positive impact. Tian and Zhu [39] used grain-sown area, labour input (indicated by agriculture, forestry, animal husbandry, and fishery), and fertiliser application as explanatory variables when studying the factors affecting food production efficiency in China. The results showed that chemical fertilisers and machinery promoted the efficiency of food production. The main factors for the improvement, the grain-sown area, and labour input have no significant impact on efficiency.

The technical efficiency measured by the DEA model was selected as the dependent variable, and farming methods, input elements, supporting services, and other factors were used as independent variables to perform the random-effects tobit model regression analysis. The statistical descriptions of the explanatory variables are presented in Table 3. The farming system is divided into five methods: no-tillage, conservation farming (reducing stubble and covering seeds), conservation farming (covering seeds), intensive farming, traditional farming, and deep farming. I constructed dummy variables for the farming system. The proportion of mechanical labour is the proportion of mechanical labour to total labour, which is used to reflect the degree of mechanisation. To explore the influence of the degree of mechanisation on technical efficiency, the square term of the proportion of mechanical labour was added to the constructed model. The proportion of hired workers indicates the ratio of the number of employees to the total labour force. The total labour force includes family and hired labour. Land cost refers to the cost of renting a unit area of land or the opportunity cost of land. Drying costs refer to the costs incurred when drying corn. Insurance premium is the cost of purchasing agricultural insurance for farms. Consultation fees refer to the expenses incurred by consulting experts in agricultural production. The addition of time-trend variables reduces the impact of time on technical efficiency. Regional dummy variables were set up, and North America was used as the benchmark group to compare regional technical efficiency differences with South America, Europe, and Africa.

**Table 3:** Model variable settings and descriptive statistics.

Variable classification	Variable name	Mean	Standard deviation
Technical efficiency		0.84	0.18
Tillage system	Deep ploughing	0.18	0.39
	No tillage	0.33	0.47
	Conservation tillage (reducing stubble and mulch seed)	0.17	0.37
	Conservation tillage (mulch seed)	0.05	0.23
	Intensive tillage	0.25	0.43
Input elements	Land cost	257.87	269.80
	Proportion of hired workers	0.74	0.36
	Proportion of mechanical labour	0.16	0.15
	The square term of the proportion of mechanical labour	0.05	0.11
Supporting service	Dry energy cost	56.48	107.37
	Insurance	9.47	29.56
	Advisory	7.24	13.24
Regional difference	South America	0.33	0.47
	Europe	0.48	0.50
	Africa	0.06	0.24
Time difference	Year	4.45	2.32

## Results and discussion

The use of the DEA model for efficiency measurement needs higher requirements for the selection of input items, and the difference in the selection of input items directly leads to differences in the results of the efficiency calculation. After the input items were determined, multiple collinear tests were performed to ensure that the selected input items were not redundant. Using Stata to test this, the results show that the input variables selected by the DEA model are all less than 10, with an average value of 2.85, indicating that the input variables selected by the DEA model are not redundant. A multicollinearity test was also performed on the explanatory variables selected by the tobit model. The results indicated that the variance expansion factors of the selected explanatory variables were all less than 10, with an average value of 2.02, indicating that there was no multicollinearity in the variable settings of the tobit model [47, 48].

### Technical efficiency and decomposition of corn production in typical farms

DEAP2.1 software was used to calculate the technical efficiency of 18 typical farms, which is shown in Table 4. The average level of technical efficiency of corn production was 0.863, and the average loss of efficiency was 13.7%. Table 3 shows that the comprehensive technical efficiency of nine typical farms is 1, indicating that half of the farms in the research group have corn production at the forefront of production, and corn production is DEA-effective. These farms are in Europe and South America, of which three are in Argentina, one is in Brazil, and the others are in the Czech Republic, Poland, Russia, Ukraine, and Uruguay. The comprehensive technical efficiency of US1215INC farms in Bulgaria, Hungary, and the US was approximately 0.5. This is because the pure technical efficiency of corn production is low, which reduces the contribution of scale efficiency. From the perspective of scale efficiency, the average level is 0.939, which is relatively high. However, half of the farms are still in a state of ineffective scale. Farms in Bulgaria, Brazil, Hungary, and South Africa are in a state of increasing returns to scale, and five farms in France and the US are experiencing diminishing returns to scale.

**Table 4:** Average corn production efficiency and return to scale of typical farms.

Country	Farm ID	Comprehensive technical efficiency	Pure technical efficiency	Scale efficiency	Return to scale
Argentine	AR330ZN	1.000	1.000	1.000	Unchanged
Argentine	AR700SBA	1.000	1.000	1.000	Unchanged
Argentine	AR900WBA	1.000	1.000	1.000	Unchanged
Bulgaria	BG7000PLE	0.405	0.521	0.777	Increasing
Brazil	BR1300MT	1.000	1.000	1.000	Unchanged
Brazil	BR65PR	0.644	0.785	0.821	Increasing
Czech	CZ4000JC*	1.000	1.000	1.000	Unchanged
France	FR110ALS	0.970	1.000	0.970	Decreasing
France	FR110VGAV	0.916	1.000	0.916	Decreasing
Hungary	HU1100TC	0.473	0.495	0.956	Increasing
Poland	PL730WO	1.000	1.000	1.000	Unchanged
Russia	RU20000BS	1.000	1.000	1.000	Unchanged
Ukraine	UA7100PO*	1.000	1.000	1.000	Unchanged
US	US1215INC	0.505	0.601	0.840	Decreasing
US	US1300ND	0.908	1.000	0.908	Decreasing
US	US700IA	0.944	1.000	0.944	Decreasing
Uruguay	UY360CEN	1.000	1.000	1.000	Unchanged
South Africa	ZA1300NW	0.764	1.000	0.764	Increasing
Average		0.863	0.911	0.939	/

Based on the two aspects of the main corn-producing countries and the production technology being at the forefront, the corn production conditions in the US, Brazil, and Argentina are analysed.

The pure technical efficiency of corn production in two typical farms in the US is 1, the scale efficiency is above 0.9, and the returns to scale are both in a diminishing stage. As the main agricultural business entity in the US, a large number of family farms have a long history of development. In 2010, the number of family farms in the US reached more than 1.9 million. On average, each family farm has a large area of arable land. The development of family farms requires a higher degree of mechanisation. Therefore, the US attaches great importance to the research and development of agricultural science and technology. The use of agricultural technology reduces labour input and saves transaction costs propagated by hired labour, which is an important reason for maintaining a high level of agricultural production efficiency [49–51]. Furthermore, the US government has always attached great importance to agricultural protection. Due to the surplus of agricultural production, the return to scale of farm corn production is in a diminishing stage. To avoid excessive production by farmers, the government has issued several compensation policies to encourage farmers to participate in fallow programs to adjust supply and protect farmers’ income [52].

The comprehensive technical efficiency, pure technical efficiency, and scale efficiency of the BR1300MT farms in Brazil and three farms in Argentina are all 1, and the return to scale remains unchanged.

Natural conditions. Brazil, Uruguay, and Argentina are all located in South America. Corn growth has more intensive water requirement. Abundant rainfall and vertical and horizontal river networks provide irrigation conditions, and corn has become the main crop in these areas.

Government support. The Brazilian government has issued several policies to support the soybean planting industry. Brazil convened soybean farmers across the country to form farm consortiums, which purchase materials for soybean planting and production in a unified manner and provide farmers with financing, product transportation, and storage services [53]. It is beneficial to reducing procurement costs, promoting the consistency of soybean product quality, and achieving large-scale mass production. Soybean varieties grown in Brazil were introduced in the US and transferred to the country for research and breeding. The new varieties cultivated by scientific research institutions based on the country’s soil and climatic conditions are more adaptable to the country’s natural conditions and more suitable for growth in the tropics and subtropics. Consequently, the yield of corn improved [54]. The Argentine government has always attached great importance to the research, development, and promotion of agricultural science and technology [55]. It has established several scientific research institutions to promote the development of agricultural science and technology, establish extension stations to provide farmers with agricultural knowledge, train the use of new technologies, promote the implementation and transformation of scientific research results [56], and establish specialised corn production areas; production specialisation has greatly improved the production efficiency of corn. The export strategy has been formulated to focus on reducing production costs and increasing output per unit area. Consequently, its competitiveness in international trade has improved [56].

### Influencing factors of corn production technology efficiency

The regression results of the tobit model are presented in Table 5.

**Table 5:** Tobit model regression results of factors affecting corn production efficiency.

Variable classification	Variable name	Regression coefficient	Standard error
Tillage system	Deep ploughing	0.14	-0.09
	No tillage	-0.01	-0.08
	Conservation tillage (mulch seed)	0.41***	-0.11
	Intensive tillage	0.36***	-0.10
Input elements	Land cost	<0.01***	0.00
	Proportion of hired workers	0.35***	-0.09
	Proportion of mechanical labour	1.05**	-0.44
	The square term of the proportion of mechanical labour	-1.25***	-0.48
Supporting service	Dry energy cost	<0.01**	0.00
	Insurance	<0.01	0.00
	Advisory	-0.004***	0.00
Regional difference	South America	-0.13	-0.12
	Europe	-0.41***	-0.11
	Africa	-0.59***	-0.14
Time difference	Year	0.03***	-0.01
	_cons	0.43***	-0.11

Note: Standard errors are in parentheses.

* p < 0.05

** p < 0.01

*** p < 0.001.

#### Regional difference

The North American region represented by the US was considered a benchmark. The regression results indicate that the average technical efficiency level of South America does not show a significant difference from that of North America. At the 1% level, the technical efficiency of Europe and Africa is significantly lower than that of North America to varying degrees. Among them, Africa, represented by South Africa, has the lowest technical efficiency. North America, represented by the US, and South America, represented by Brazil and Argentina, have the highest maize production efficiency. The US, Brazil, and Argentina are the main corn producers and exporters. In addition to having a unique natural resource endowment, government agencies have invested heavily in science and technology and issued several policies to protect the interests of farmers and promote and guarantee the effective production of corn [56].

#### Time difference

At the 1% significance level, the time variable had a positive effect on technical efficiency. This shows that over time, the technical efficiency of agricultural production is constantly increasing, and the level of agricultural technology is constantly improving. The advancement of agricultural production technology relies on economic growth. In the exogenous technological progress growth model, the technological factor is regarded as a function of time.

#### Tillage system

The regression coefficients of deep tillage and no-tillage did not pass the significance test, indicating that the technical efficiency of deep tillage and no-tillage did not show obvious differences compared with conservation tillage (reducing stubble and covering seeds). At a significance level of 1%, seed mulching and intensive farming have higher technical efficiency than conservation tillage, and seed-covering farming modes have higher technical efficiency. Covering seeds can replace fallow in summer, protect soil organic carbon, and have beneficial effects on maintaining soil fertility and improving soil quality, thereby effectively disposing biological waste [57]. Conservation tillage integrated mulch technology can effectively coordinate crop yield, water consumption, and reducing carbon emissions, which can increase the water use efficiency of corn and improve corn production efficiency [58].

#### Supporting service

The input of the drying fee reflects that typical farms dry harvested corn. However, not all production entities dry corn, but the harvested corn has a high moisture content. If stored improperly, the corn will deteriorate and produce mildew, resulting in food waste and economic losses [59]. In the regression results, the drying fee has a significant promoting effect on the improvement of technical efficiency, indicating that the drying treatment of corn significantly reduced the loss of harvested fruits, promoted agricultural efficiency, and improved corn production efficiency.

The farm insurance premium did not pass the significance test in the regression results, indicating that agricultural insurance did not fully promote the production of planting entities. Due to the risks of agricultural production that are difficult to control, the agricultural insurance market system is unsophisticated [60], the implementation of insurance is arbitrary, and the protection of agricultural production is not enough; therefore, the interests of farmers have not been effectively protected [61]. Expert consulting fees have a significant inhibitory effect on corn production efficiency at the 1% level. On the one hand, the methods used to collect farmers’ knowledge are flawed, resulting in inaccurate or incomplete information. The use of inaccurate information or misunderstandings between farmers and scientists result in the development and extension of unprofitable, unsustainable or inappropriate management recommendations [62]. On the other hand, a small number of experts are far from the reality of agricultural production and fail to propose practical solutions to farmers. Moreover, farmers are restricted by their level, unable to achieve effective docking between farmers and experts, and have not played a role in improving agricultural production efficiency [63].

Regarding input factors, at a significance level of 1%, land cost has a positive impact on technical efficiency, but the impact is relatively small. This indicates that when the cost of land input factors increases, it will curb the input of land factors, which in turn will increase the utilisation rate of existing land and promote an increase in land productivity. They cope with the increase in the cost of production materials by increasing the output per unit area of land and promoting the improvement of production technology. The regression coefficient of the proportion of hired workers is significantly positive at the 1% level, indicating that it is difficult to improve technical efficiency by relying solely on family labour. The increase in the proportion of hired labour in labour input has a positive effect on the development of agricultural production. Additionally, as agricultural production reaches a certain stage, the demand for labour will inevitably increase. The limited family labour force has led to a large demand for hired labour, which has met the expansion of agricultural production and stimulate technical efficiency.

At a significance level of 5%, the proportion of mechanical labour can promote technical efficiency. Due to the continuous increase in labour costs, the main body of production has increased the use of machinery [64], shortened the operation time of unit agricultural production, substantially increased labour productivity [65], and improved corn production efficiency. However, at the 1% significance level, the regression coefficient of the square term of the proportion of mechanical labour is negative, indicating that there is an inverted U-shaped relationship between the proportion of mechanical labour and technical efficiency. When the proportion of mechanical labour exceeds the critical value, continuing to invest in machinery will lead to a decline in technical efficiency, which is due to the use of machinery restricted by topographical characteristics and agricultural production conditions [66]. Plain areas are easier to mechanise than hilly areas. Small machinery can be used in hilly areas. Excessive machinery investment leads to low agricultural production efficiency and economic losses. The use of machinery also has requirements for the extent of the entire land. It is difficult for these fragmented lands to implement large-scale mechanised production. Agricultural operations still rely mostly on human labour. The proportion of machinery investment is too high, and it is difficult for them to be effective, resulting in technical inefficiency.

## Conclusions

The main conclusions of this study are as follows: first, the overall level of technical efficiency of corn production on family farms in Argentina, the US, Ukraine, and other countries is at a relatively high level and is at the forefront of production technology. However, some farms are affected by the low efficiency of pure technology. Technical inefficiency reached 50%, and the average loss of technical efficiency was 13.7%. Second, among the five farming modes, seed-covering, intensive farming, and conservation farming have higher production efficiency than no-tillage and deep tillage. The increase in the cost of production factors of land and labour forces has caused the continuous improvement of technology, thereby increasing the efficiency of corn production. Third, corn drying can promote production efficiency. Farm insurance premiums and expert consultation fees have not played a role in stabilising agricultural production and promoting crop management. The transformation of the economic benefits of agricultural production equipment services is still lacking. Finally, the input and use of machinery are within a certain threshold, which improve labour productivity and promote the development efficiency of corn planting agriculture. However, if the mechanical input exceeds the turning point, it will cause redundancy in the input of elements, resulting in waste and loss of efficiency.

The findings of this study are as follows. First, to achieve the goal of improving corn production efficiency, reducing biological waste, and realizing green and economical production, it is necessary to innovate the production and farming mode of corn, promote the diversification of production methods, and develop a protection farming method that combines with bean crops. Second, improving and refining the construction of agricultural insurance regulations and systems, effectively protecting farmers’ interests, and sharing the risks faced by agricultural production for farmers, are all conducive to improving the efficiency of food production and its sustainability. Third, it is high time to promote the landing and transformation of agricultural scientific research results, strengthen the training and support of scientific research institutions, experts and scholars to production entities, and earnestly take farmers as the main resource for agricultural production ideas. Fourth, it is imperative to consider to promote land circulation and reduce fragmented land to create conditions for mechanised agricultural production and provide basic guarantees for modern agricultural production. Simultaneously, various types of machinery should be developed as well as a technical strategy that combines large-scale and small-scale machinery. For areas where it is difficult to operate large-scale machinery, small-scale agricultural machinery with characteristics of adapting to local conditions should be considered. Finally, strengthen the construction and improvement of the agricultural service system and provide farmers with various forms of information services so that agricultural products can better meet the market demand. At present, there is a large gap between socialised service funds and unreasonable use. Agricultural socialised services must be considered with high importance to effectively serve both farmers and agriculture, ensure agricultural production, and improve economic benefits.

## Supporting information

S1 Table Corn production input and output indicators in typical farms for 2012–2019. (XLSX)

S2 Table Model variable settings and descriptive statistics. (XLSX)
